# Technology-Based Stepped Care to Stem Transgender Adolescent Risk Transmission: Protocol for a Randomized Controlled Trial (TechStep)

**DOI:** 10.2196/18326

**Published:** 2020-08-13

**Authors:** Cathy J Reback, Joshua A Rusow, Demetria Cain, David Benkeser, Sean Arayasirikul, Lisa Hightow-Weidman, Keith J Horvath

**Affiliations:** 1 Friends Research Institute, Inc Los Angeles, CA United States; 2 UCLA Center for HIV Identification, Prevention, and Treatment Services University of California, Los Angeles Los Angeles, CA United States; 3 PRIDE Health Research Consortium Hunter College City University of New York New York, NY United States; 4 Department of Biostatistics and Bioinformatics Rollins School of Public Health Emory University Atlanta, GA United States; 5 Department of Pediatrics Division of Developmental Medicine University of California, San Francisco San Francisco, CA United States; 6 Trans Research Unit for Equity San Francisco Department of Public Health San Francisco, CA United States; 7 Institute for Global Health and Infectious Diseases The University of North Carolina at Chapel Hill Chapel Hill, NC United States; 8 Department of Psychology San Diego State University San Diego, CA United States

**Keywords:** HIV, acquired immunodeficiency syndrome, transgender, technology, pre-exposure prophylaxis, mobile phone

## Abstract

**Background:**

Transgender youth demonstrate significantly higher rates of engagement in sexual risk behaviors relative to their cisgender or gender-conforming counterparts, including high rates of condomless anal intercourse and engagement in sex work. In addition, transgender youth experience increased physical or sexual abuse, victimization, substance use, mental health disorders, incarceration, and homelessness. Owing to these syndemic health disparities, transgender youth are at substantially increased risk of HIV infection.

**Objective:**

This protocol aims to describe a randomized controlled trial (RCT), Adolescent Medicine Trials Network 160 TechStep (N=250), which assesses the differential immediate and sustained effects of each of 3 conditions (text messaging, WebApp, or information-only control) for reducing sexual risk behaviors and increasing pre-exposure prophylaxis (PrEP) uptake among high-risk, HIV-negative transgender youth and young adults (aged 15-24 years).

**Methods:**

Participants will be recruited through web-based (targeted social media sites and apps) and offline (print ads and flyers) advertisements, peer and clinic referrals, and street- and venue-based outreach, and by contacting potential participants who have requested contact for future studies. Participants will be randomized into 1 of the 3 conditions: (1) text messaging, (2) WebApp, or (3) information-only control for 6 months. Assessments will occur at baseline and at 3, 6, and 9 months. Participants who do not show improvements in sexual risk or PrEP uptake at the 3-month assessment will be rerandomized to receive weekly electronic coaching (eCoaching) sessions in addition to their assigned text messaging or WebApp intervention, or remain in the original text messaging or WebApp intervention using a 2:1 ratio. Participants originally assigned to the information-only condition are not eligible for rerandomization.

**Results:**

Funding for TechStep was awarded in June 2017. Phase 1 was approved by the Institutional Review Board (IRB) in April 2018. Recruitment began in November 2018 for phase 1, the formative phase. Initial phase 2 IRB approval came in June 2019. The data collection for phase 2, the RCT, is expected to be completed in April 2021. As of March 2020, 54 participants have been enrolled in TechStep. The final results are anticipated in May 2021.

**Conclusions:**

By providing culturally responsive, technology-based interventions, TechStep aims to improve sexual health outcomes among HIV-negative transgender youth and young adults at high risk of HIV. TechStep will evaluate the efficacy of technology-based interventions for reducing HIV sexual risk behaviors and increasing PrEP initiation, adherence, and persistence. The suite of technology-based interventions developed in TechStep, and assessed for efficacy in a 3-condition RCT, represents an important advancement in intervention science toward developing tailored and scalable interventions for transgender youth and young adults.

**Trial Registration:**

ClinicalTrials.gov NCT04000724; http://clinicaltrials.gov/ct2/show/NCT04000724

**International Registered Report Identifier (IRRID):**

DERR1-10.2196/18326

## Introduction

### Background and Study Objectives

National evidence suggests that as many as 8% of youth in the United States self-identify as *transgender*, *gender nonconforming*, or *other gender* (hereafter, trans) [1] and that trans youth face a *health syndemic*, in which a set of reinforcing structural, cultural, and behavioral factors place them at a dramatically increased risk for negative physical, social, and mental health outcomes [2-6]. For example, trans youth demonstrate significantly elevated rates of sexual risk-taking relative to their cisgender or gender-conforming peers [4,7-9], including rates of condomless anal intercourse ranging from 27% to 59% and rates of engagement in sex work ranging from 24% to 75% [9,10]. Increased rates of sexual risk behaviors among trans youth have been associated with the population’s increased experiences of physical or sexual abuse and victimization, mental health disorders, incarceration, and homelessness [4,9,11] as well as substance use, sex work, and substance use during sex [4,9,12]. As a result of this health syndemic, trans youth, especially racially and ethnic minority trans youth, are at a substantially increased risk for HIV infection as well as other sexually transmitted infections (STIs) relative to their cisgender youth counterparts [10,12,13]. Despite these needs, there is persistent evidence of a lack of culturally competent care and inadequate training of traditional health care providers [14,15]; the difficulties trans youth report accessing traditional health care [8,16] and the frequent reports by trans youth of prejudice or discrimination when receiving traditional health care [3,5] demonstrate the critical need for avenues of sexual health information and interventions that extend beyond traditional *brick-and-mortar* services and cater to the special needs of trans youth.

All adolescents, including trans youth [17], demonstrate the frequent use of text messaging and mobile internet-enabled technology to seek protective sexual health information [18]. Trans youth explicitly cite mobile phones as critical tools in their ability to engage with sexual health information, as text messaging conversations, mobile apps, and other mobile health delivery modalities provide portals where trans youth report feeling comfortable seeking health-related knowledge specific to trans individuals (eg, hormone therapy, pubertal suppression [19]), adopt and express new and different identities, and connect to other trans youth to seek information and resources [17,20,21]. Among mobile intervention modalities, text messaging and smartphone apps have been identified as particularly well suited to the needs of trans populations, whose gender expression may serve as an obstacle to standard in-person treatment [22].

A recent systematic review revealed that only 18% of mobile phone–based HIV prevention or care interventions provide any information tailored to the lesbian, gay, bisexual, transgender (LGBT) populations, and none were tailored specifically to trans individuals [23]. Thus, there is a clear need to develop effective prevention interventions that meet the needs of trans youth and young adults and are developed specifically for this population.

### Objectives

The *TechStep* study will address a critical gap in the scientific advancement of technology-based interventions for trans youth by (1) creating the first technology-based trans youth–specific HIV prevention intervention optimized for mobile phone delivery and (2) employing the 2 intervention delivery modalities (ie, text messaging and mobile WebApp) identified as the most promising for use among trans youth and young adults. The suite of technology-based interventions developed in *TechStep* and assessed for efficacy in a three-condition randomized controlled trial (RCT) represents an important advancement in intervention science toward developing tailored and scalable interventions for trans youth.

## Methods

### Research Aims

As part of the University of North Carolina/Emory Center for Innovative Technology (iTech) [24], we proposed to test the efficacy of Adolescent Medicine Trials Network (ATN) 160 *TechStep* for trans youth and young adults. In this 4-year study, 250 high-risk trans youth and young adults aged between 15 and 24 years will be randomized to receive text messaging, a WebApp (ie, a website that is optimized for display on smartphones), or an information-only control intervention for 6 months, with assessments occurring at baseline and at 3, 6 and 9 months. Participants who do not show improvements in sexual risk or pre-exposure prophylaxis (PrEP) uptake at the 3-month assessment will be randomized to receive weekly electronic coaching (eCoaching) sessions in addition to their assigned text messaging or WebApp intervention, or remain in the original text messaging or WebApp intervention using a 2:1 ratio. The primary endpoint is reduced sexual risk behaviors or PrEP uptake, adherence, and persistence at month 6 and sustained through month 9.

The aims of the research include the following:

1. Primary aim 1: conduct formative research to develop stepped care (text messaging, WebApp, and eCoaching) interventions and refine iterations through input from focus groups with trans youth and young adults at the 4 study sites (Houston, Texas; Los Angeles, California; New York, New York; and Philadelphia, Pennsylvania) and with a trans-specific youth advisory board (YAB).
2. Primary aim 2: in a 3-condition RCT (N=250), assess the differential immediate and sustained effects of a low-intensity information (*Info*) condition compared with a text messaging stepped care intervention (text messaging plus step to eCoaching for youth and young adults with continued high risk) condition compared with a WebApp stepped care intervention (WebApp plus step to eCoaching for youth and young adults with continued high risk) condition for reducing sexual risk behaviors and increasing PrEP uptake among high-risk, HIV-negative trans youth (15-24 years old):
  Hypothesis 1a: there will be significantly greater reductions in sexual risk behaviors among those in the text and WebApp conditions compared with the low-intensity info condition.
  Hypothesis 1b: there will be significantly greater uptake of PrEP among those in the text and WebApp conditions compared with the low-intensity info condition.
3. Secondary aim 1: determine the added benefit of text messaging plus eCoaching (text+eCoaching) versus text messaging alone and of WebApp plus eCoaching (WebApp+eCoaching) versus WebApp alone to reduce sexual risk behaviors and increase PrEP uptake.
4. Secondary aim 2: assess the differential immediate and sustained effects of low-intensity information compared with text messaging only compared with WebApp only for reducing sexual risk behaviors and increasing PrEP uptake.
5. Secondary aim 3: determine the impact of structural-level (eg, transphobia, housing insecurity, educational attainment, access to health care) and individual-level (eg, identity formation, gender transition, gender expression, stigma, discrimination) trans-specific factors as moderators of intervention outcomes.
  Hypothesis 2: structural- and individual-level trans-specific factors will moderate intervention outcomes, such that participants who report higher amounts and degrees of these factors will require more intensive intervention steps (ie, eCoaching).

### Ethics Statement

The Institutional Review Board (IRB) at the University of North Carolina, Chapel Hill, NC, is the IRB on record for all participating institutions and subject recruitment venues (SRVs) participating in the study. The study procedures were approved by the University of North Carolina IRB 18-0519. A waiver of parental consent was obtained for participants aged 15 to 17 years. The study was registered as a clinical trial (Clinical Trials #NCT04000724).

### Interventions

#### Text Messages

The theoretical construct for the text message intervention was based on 3 proven theories of behavioral change, and the text messages will be equally distributed across the 3 behavioral change theories: Social Cognitive Theory, Health Belief Model, and Social Support Theory.

##### Social Cognitive Theory

The Social Cognitive Theory posits interactive causal relationships among personal determinants, behavior, and environmental influences [25,26]. Effective HIV prevention interventions must increase individuals’ self-efficacy and guide them in developing self-regulation skills, offering practice and feedback opportunities, and engaging social support resources to maintain prevention behavior.

##### Health Belief Model

The Health Belief Model asserts that individuals’ beliefs regarding threats to their health, and that specific health behaviors can reduce these threats, predict their likelihood of engaging in protective health behaviors [27]. The Health Belief Model is most effective when messages regarding threats and beliefs are culturally appropriate to the specific target population.

##### Social Support Theory

According to the Social Support Theory, social support encompasses instrumental, emotional, and informational assistance provided by members of one’s social network. These forms of social support have been shown to mediate the relationship between stressful events and health outcomes [28,29].

##### Text Message Delivery

Participants assigned to the *TechStep* text message intervention will receive 2 cycles of the 90-day text messaging intervention, that is, participants will receive the same intervention twice, once from 1 to 90 days and then again from 91 to 180 days. During the intervention period, participants will receive 3 scripted, theory-based, trans-specific text messages per day (a total of 270 text messages that will be repeated once). Text messages will be transmitted through gradual automation administration every day, including weekends, in real time, within a 10-hour period. Thus, participants will receive a text message approximately every 5 hours starting at either 9:00 AM or noon local time (eg, at noon, at 5:00 PM, and at 10:00 PM). Timing was determined based on findings from the 3 previous text messaging studies conducted by the Protocol Co-Chair [30-32] and supported by feedback from focus group participants. Participants will select whether to start receiving messages in the morning, starting at 9:00 AM, or in the afternoon, starting at noon. The text messages were specifically scripted, with input from the focus group participants and YAB members, for HIV-negative trans youth and young adults who are at risk of HIV infection. The automated text message delivery system was developed specifically for this study by Dimagi [33], a digital software company that specializes in global health technology.

Table 1 illustrates how the text message library was developed to have both a theoretical foundation and be transculturally responsive.

**Table 1:** Sample of scripted text messages by behavioral change theory.

Social Support Theory			Health Belief Model		Social Cognitive Theory		
Informational support	Emotional support	Instrumental support	Health threat	Health behaviors to reduce threat	Awareness of health risks	Self-regulation skills	Self-efficacy
Think you might have been exposed to HIV? Start PEP^a^ within 72 hours**,** and taking it every day for 28 days will keep you protected	Your body and soul are beautiful. See a health care provider	PrEP^b^ exists, take advantage of it!	Untreated STIs^c^ can steal your beauty	Take care of yourself and your trans community. Check out resources at (hyperlink)	Lead your community by example, take your PrEP daily!	Taking care of yourself is loving your trans body	Know your health info, be empowered, see your doctor
Get the “T” on STI info and testing call (phone number)	Trans Pride is taking care of yourself	Seen your partner lately? See your doctor, too	We love you, don’t be a statistic, take your PrEP!	Be smart, safe**,** and sexy	Protect your cute trans body, see your doctor	Had unprotected sex? No shame, get on PrEP	Nothing compares to you, you can be safe

^a^PEP: post-exposure prophylaxis.

^b^PrEP: pre-exposure prophylaxis.

^c^STI: sexually transmitted infection.

#### WebApp

**Figure 1:**
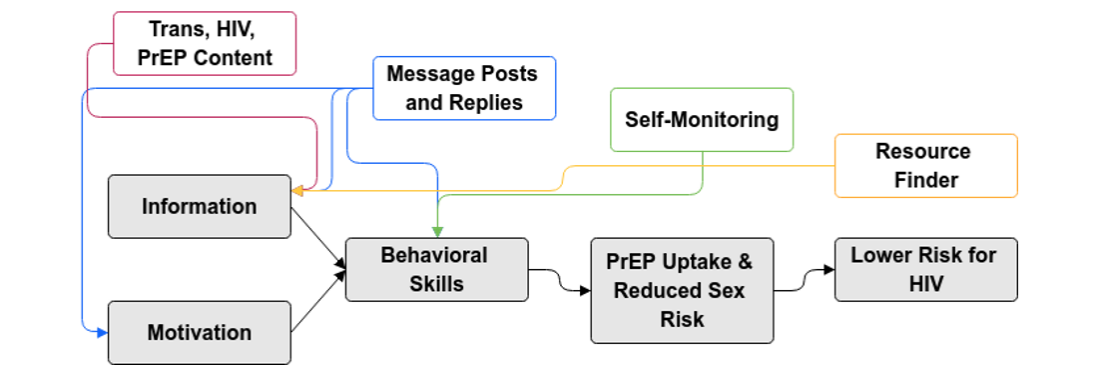
TechStep WebApp components and the Information, Motivation, Behavior model. PrEP: pre-exposure prophylaxis.

The theoretical construct for the WebApp intervention was based on the Information Motivation Behavior (IMB) model of behavioral change (Figure 1).

##### IMB

The IMB model proposes that health behavior and behavior change results from being well and accurately informed, having the personal and social motivation to engage in the behavior, and having the appropriate behavioral skills and self-efficacy to use them [34-36]. The associations between core WebApp intervention components (described in detail below) and the IMB model components are shown in Figure 1. The IMB model has been used to predict risky sexual behavior among adolescents in Los Angeles, California [37], and has been used as the theoretical basis of adolescent risk reduction interventions [38].

##### WebApp Development

The WebApp intervention will be developed as a safe digital space for sharing information and helping trans youth and young adults feel empowered and supported to make healthy sexual health choices. To be available to answer questions and enforce community standards (eg, no hostile exchanges), the WebApp will be moderated by research staff (primarily graduate-level students) trained by the study Protocol Co-Chair on how to identify and respond to problematic posts. Moderating includes reading through posted comments on the wall each day and identifying posts that are concerning (eg, suicidal ideation, pleas for assistance, and potentially hostile comments to other users). Posts made by participants are not delayed or held until *cleared* for posting. Rather, the moderator reviews posted material and acts accordingly. This is to retain the immediacy of posting, which users of social media largely expect. The following are the core components of the WebApp intervention.

###### Message Posting and Receiving

The WebApp homepage will consist of an interface for participants to asynchronously interact with one another through message posting (Multimedia Appendix 1). Unlike widely used social networking platforms such as Facebook, participants will view all posts on one shared feed (vs individual feeds or direct messaging). Other users may comment on a post as well as use reaction buttons (eg, thumbs up, combined LGBT and trans flag). Message posting is the primary social support component of the intervention as it allows participants to directly and voluntarily interact with one another in a similar manner as a face-to-face peer support group.

###### HIV Prevention and Trans-Specific Information

Staff members who are a part of the community wrote brief informational pieces of content, called *Tips*, on the WebApp, covering a broad range of topics including HIV prevention, sexual health, PrEP, and transphobia. Youth in the WebApp intervention condition will receive approximately 3 tips each day. Each tip contains a combination of written content and a video, meme, or graphic. The study staff created approximately 250 tips in total, with approximately three-fourth dedicated to specific content areas (eg, PrEP and sexual health) and the remaining considered *grab bag* tips related to other important topics to the community. Tips may be favorited and reviewed later or explored through tags that will display all the tips related to that tag.

###### Self-Monitoring

Participants will have the ability to self-monitor one or more behaviors under the *Tracker* tab. Participants can create a new tracking behavior by inputting the behavior they would like to track, with *hormones* and *PrEP* suggested, but also offering the ability to key in the behavior of their choice. Next, participants are asked how they would like to be reminded (through SMS or on the app) and the frequency of the reminder (daily or weekly). Once they set up the behavior to be tracked, they will be able to indicate whether they performed that behavior that day. Underneath, a monthly calendar will be displayed that reflects the frequency with which they reported the behavior and the ability to toggle between the different behaviors they are currently tracking.

###### Resource Locator

Participants will be able to search for local trans-specific resources in their area through the *Resources* tab. Resources (eg, HIV testing, PrEP provision, housing assistance) were identified through web-based searches and by asking local SRV contacts to provide a list of trans-specific resources. Next, the study staff called each agency to confirm that they were still in operation and that they serve transgender persons as part of their services. Once confirmed, the study staff entered information about the resources in the WebApp database, including hours of operation, testing services, and address, which can use the location of the phone to show the distance from the participant. Participants can rate (using a five-point star system) and comment on the resource for others to view as well as suggest new resources for the database (Multimedia Appendix 2).

###### Weekly SMS Engagement Message

All participants will receive a weekly SMS (text) message that prompts and encourages them to visit the *TechStep* WebApp. SMS messages are designed to engage youth with different aspects of the site by providing a link within the message that will take youth directly to the WebApp.

###### Game Mechanics

The WebApp uses points that accumulate as youth use intervention components to reinforce engagement with the site. As points accumulate, youth move through higher levels (ie, *levelling up*) during the intervention period, which unlocks new features of the site (eg, new avatar choices and color theme choices) when a new level is achieved. Points are earned through posting on the WebApp feed (wall), responding to other users’ comments, setting new goals, clicking on a tip, and other actions that may be taken in the WebApp. Youth will be able to view the number of points and their current level as part of their profile.

#### eCoaching

Participants in the text or the WebApp conditions who do not reduce sexual risk behaviors or who self-report a recent STI diagnosis and do not initiate PrEP or adhere to PrEP during the first 3 months of those interventions will be rerandomized to additionally receive eCoaching. The eCoaching intervention integrates theoretical constructs of Motivational Interviewing (MI) and Cognitive Behavioral Therapy (CBT) to assist trans youth and young adults in establishing health behavior goals, discussing facilitators and barriers to behavior change, and providing behavioral skills to enhance goal attainment [39].

##### MI

MI is a style of communication fostering collaboration and goal setting to build personal motivation for behavior change [40]. MI techniques build upon the essential elements of partnership, acceptance, compassion, and evocation through 4 different MI processes. A session commences with *engagement* to build a rapport and therapeutic alliance. *Focusing* allows the participants to discuss their own goals and priorities as it relates to the target behavior. *Evoking* elicits change talk by exploring a participant’s thoughts on behavior. Finally, *planning* allows for specific goal setting, summarizing, identifying potential barriers, and discussing options for overcoming barriers. eCoaches move participants through these processes with MI techniques of asking open-ended questions, using simple and complex reflections, sustaining change talk, and summarizing.

##### CBT

CBT is an action-orientated treatment that addresses maladaptive cognitive beliefs and behaviors [41]. It is based on the premise that all behavior is learned and can be *unlearned* with the introduction of new behavioral skills, including problem solving, assertiveness and communication training, self-monitoring, environmental control, distress tolerance, and how thoughts and feelings affect behavior.

eCoaching sessions are conducted through a Zoom portal (Zoom Video Communications Inc) to have a common meeting place for video conferencing and electronically sharing intervention activity materials. Participants will be asked to join eCoaching sessions through their mobile device or desktop. Sessions are 30 to 40 min long and held weekly on an agreed-upon day and time.

Participants rerandomized to eCoaching are virtually introduced to their eCoach during the 3-month assessment appointment, called the First Contact session. The First Contact session is a 20-min mini session during which the participant meets their eCoach, learns about the eCoaching intervention, establishes rapport, introduces the functional assessment, and establishes an agreed-upon day and time for subsequent weekly virtual sessions. There are up to 8 sessions of content available to occur over a 12-week period. Sessions 1 to 4 are core sessions: session 1: Planning for My Plan; session 2: You Getting to Know You; session 3: Intimacy and Communication; and session 4: Getting PrEPared. Sessions 5 to 8 are optional and are based on answers to the functional assessment and subsequent collaborative treatment planning. Session 5: Keeping It Cool; session 6: Alcohol and Drugs; session 7: Checking In; and session 8: You Don’t Have to Do It Alone. The functional assessment is a 20-item survey completed between first contact and session 1 with yes, no, and maybe response options. Endorsing certain items (yes or maybe) map onto recommended modules for sessions 5 to 8 and allows the eCoach to work with the participant during the session 1 collaborative treatment plan to decide which sessions would be most beneficial.

eCoaching is facilitated by highly trained transgender and cisgender paraprofessionals centrally located at Hunter College. All eCoaches attend 5 days of MI, CBT, and protocol training conducted by a Motivational Interviewing Network of Trainers–certified trainer followed by mocking and supervision for session clearance. Fidelity is monitored through the completion of the MI coach ratings scale by the clinical supervisor and weekly supervision sessions [42].

#### Information Control Condition

The information control condition is a static website that comprises 6 pages: *Welcome*, *HIV Information*, *PrEP Information*, *Trans Information*, *Trans Resources*, and *Study Sites Contact Information*. The *Welcome* page introduces participants to the site and highlights values around trans empowerment and trans rights. The *HIV Information* page provides information on a number of HIV topics, including HIV transmission, HIV and oral sex, HIV risk in pregnancy, condom use, undetectable=untransmittable, HIV and other STIs, HIV and substance use, and HIV stigma. The *PrEP Information* page includes information about PrEP usage, methods of acquisition, and web-based and local PrEP resources in the 5 study site cities: Boston, Massachusetts; Houston, Texas; Los Angeles, California; New York, New York; and Philadelphia, Pennsylvania. The *Trans Information* page provides subsections on language, definitions, and trans history. The *Trans Resources* page provides local resources for trans youth and young adults in the 5 study site cities. Those randomized into the information control condition remain in the control condition throughout the 6-month intervention period and are not eligible for the 3-month rerandomization or the eCoaching intervention.

### TechStep Study Design

The *TechStep* study will be evaluated in a randomized controlled efficacy trial. There are 2 phases of the *TechStep* study.

#### Phase 1: Formative Research and Community Input

A total of 2 trans-specific YABs will be convened throughout the life of the study. There is a physical YAB in Los Angeles, and a virtual cross-site YAB conducted via telecommunication comprised members from the Houston, New York, and Philadelphia SRVs. The YABs will meet at least biannually and will provide feedback on all aspects of the *TechStep* study.

We conducted 7 focus groups at 4 SRVs (n=34); 2 were held in Los Angeles (n=11), 2 in Houston (n=7), 2 in New York (n=11), and 1 in Philadelphia (n=5). The New York and Philadelphia focus groups gave feedback on the text messaging intervention, whereas the Los Angeles and Houston focus groups gave feedback on the WebApp. In each city, the focus groups were stratified by age, 1 focus group consisting of participants between the ages of 15 and 20 years and the other focus group consisting of participants between the ages of 21 and 24 years (only 1 focus group, with participants aged 21-24 years, occurred in Philadelphia). This stratification ensured that the perspectives of both youth and young adults were explored. Focus groups were transcribed verbatim, and content analysis was conducted using the iTech Analytic Core. Feedback from the focus groups was used to inform phase 2.

The focus group inclusion criteria were as follows: (1) self-identification as trans feminine, trans masculine, or gender nonconforming or birth sex and current gender differ; (2) self-reported 15 to 24 (inclusive) years of age at screening; (3) report any sex with another person in the previous 12 months; (4) self-reported HIV-negative serostatus; (5) live in the area and have the availability to attend the group; (6) have a mobile device with SMS and internet access capabilities; and (7) proficient in English as determined by study staff (as the focus groups were conducted in English).

All persons who screened eligible for a focus group were guided through the informed consent process on the day of the focus group. Following informed consent, participants completed a brief paper-and-pencil focus group participant survey (eg, additional demographics and technology use survey).

Focus groups were conducted by research staff and lasted approximately 120 min. Food was served in each group. Participants were compensated for their time once the focus group ended.

#### Phase 2: RCT to Test the Efficacy of TechStep

**Figure 2:**
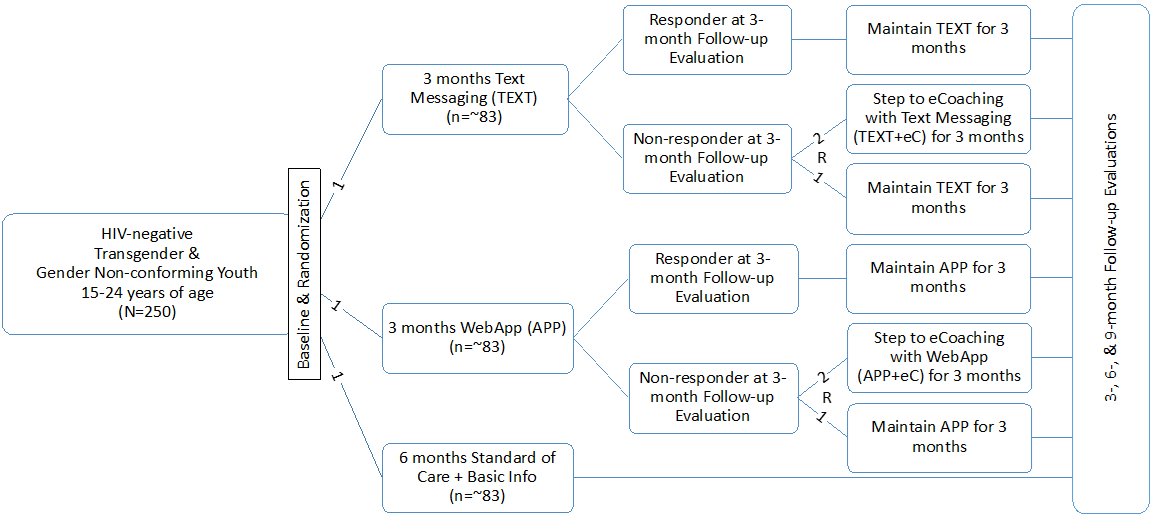
TechStep study design. eCoaching: electronic coaching.

We propose to enroll 250 trans youth and young adults in the RCT (approximately n=83 for text, n=83 for WebApp, and n=83 for information control; Figure 2). Trans youth and young adults will be recruited from 5 SRVs (Baylor College of Medicine Adolescent Medicine Trials Unit in Houston, Children’s Hospital of Philadelphia, Children’s Hospital Los Angeles, the PRIDE [Promoting Resilience, Intersectionality, Diversity, and Equity] Health Research Consortium in New York City, or the Fenway Institute in Boston) to participate in a three-condition RCT to determine immediate and sustained effects of the text intervention versus the WebApp intervention compared with an information control condition (*Info* condition).

All participants will receive one of the interventions for 6 months, with assessments occurring at baseline and every 3 months thereafter through Month 9. Trans youth and young adults randomized to either of the technology-based intervention conditions (text or WebApp) will be evaluated at the three-month follow-up assessment time points to determine whether they remain at the current level of intervention or whether they are eligible for rerandomization to also receive eCoaching sessions in addition to their originally assigned intervention (ie, text+eCoaching or WebApp+eCoaching). At the first follow-up assessment time point, information about their sexual behavior in the past 3 months and whether they began or stopped using PrEP will be used to determine whether they require a more intensive intervention approach. Participants who do not demonstrate intervention responsiveness at the 3-month follow-up assessment will be rerandomized, in a 2:1 ratio, to either add eCoaching to their original intervention (ie, text+eCoaching or WebApp+eCoaching) or remain in their original intervention (ie, text or WebApp). This rerandomization will allow for a comparison of intervention effects between the technology-based interventions (ie, text or WebApp) plus eCoaching with the technology-based intervention alone. The control condition will receive the same information-only intervention for the entire 6-month intervention period.

The RCT inclusion criteria were as follows: (1) self-identify as trans feminine, trans masculine, or gender nonconforming or birth sex and current gender differ; (2) aged 15 to 24 years (inclusive) at the enrollment visit; (3) self-report vaginal or anal sex (either insertive or receptive, excluding sex toys) with another person in the previous 12 months; (4) negative HIV rapid test; (5) live in the area and be available to meet with research staff at 1 of the 5 SRVs; (6) have a mobile device with SMS and internet access capabilities; and (7) be able to read and speak English (as the interventions will be built in English).

### Study Recruitment

The following recruitment strategies will be used to ensure diversity of the participants enrolled:

1. Web-based: web-based banner advertisements will be placed through geomapping on websites and social media in the SRV cities. Digital flyers will be distributed to community leaders to disseminate to their email distribution lists.
2. SRV and community-based reach: flyers and posters will be distributed in SRVs and other community-based organizations and clinic settings that cater to trans youth and young adults.
3. Peer long-chain referral: participants who screen eligible will be asked to refer friends who are also trans youth and young adults.
4. Clinic: clinic-based recruitment may include reviewing the medical charts of existing patients for potential eligibility or referrals from other providers in the clinic.
5. Previous participants: participants from other studies who have previously given consent to be contacted for future research may also be contacted directly.
6. Print media: advertisements may be placed in print media identified through the YABs.
7. Street- and venue-based outreach: research assistants will use a semistructured time-space sampling methodology to conduct street- and venue-based outreach identified through the YABs [43].

### Screening

All potential participants will complete a web-based screening survey to obtain consent or assent to be screened and verify all inclusion criteria. Screening may occur on the same day as enrollment (or beforehand, if screening on the web). The web-based screening survey will begin with a script to explain the purpose of screening and clarify that if they are eligible, they will be invited to participate in the study. The script will also provide general information about *TechStep*, the nature of the screening questions and related potential risks, the approximate length of the screening (approximately 5 min), the confidentiality and use of the screening information, the ability to skip any question or withdraw at any time, and contact information of key study personnel. Participants who agree to voluntarily complete the screening procedure will electronically indicate their agreement and then participate in the screening survey. SRV staff may also screen participants in person or over the phone using a web-based assessment.

### RCT Enrollment

All persons who screened eligible for the study will be guided through the informed consent process on the day of their enrollment appointment in person at one of the SRVs. Enrollment procedures will last approximately 120 min and consist of informed consent or assent; the baseline audio computer-assisted self-interview (ACASI) survey; HIV, STI, and drug screen testing; and Mitra blood analysis for detection of PrEP concentrations among those who self-reported PrEP use (Table 2). Research staff will stop enrolment if any potential participants appear confused or otherwise unable to complete the informed consent process. Following the baseline measures, the participant will be randomized into the text condition, the WebApp condition, or the information-only (ie, *Info*) condition, after which they will be considered enrolled. After randomization, participants will receive more information about the condition they are randomized to (ie, the frequency of text messages, how to use the WebApp, or how to access the information website). Participants will be compensated with the following US dollar cash or cash equivalent: US $50 at the enrollment visit, US $55 at the 3-month follow-up visit, US $60 at the 6-month follow-up visit, and US $65 at the 9-month follow-up visit.

**Table 2:** Study outcome variables and data collection schedule.

Variables	Baseline	3-month assessment	6-month assessment	9-month assessment
Rapid HIV test	✓^a^	✓	✓	✓
STI^b^ tests^c^	✓	✓	✓	✓
Blood microsampling for PrEP^d,e^	✓	✓	✓	✓
Sexual behavior	✓	✓	✓	✓
Demographics: date of birth, race and ethnicity, sex assigned at birth	✓	—^f^	—	—
Demographics: gender identity, sexual identity, education, employment status, health insurance, family income, housing stability, history in the criminal justice system	✓	✓	✓	✓
Health care utilization	✓	✓	✓	✓
Gender congruence, stress, and resilience	✓	✓	✓	✓
Substance use	✓	✓	✓	✓
Mental health	✓	✓	✓	✓
Technology adaptation and use	✓	✓	✓	✓
Intervention ease of use, acceptability, and satisfaction	—	✓	✓	—
User engagement	—	✓	✓	—

^a^Outcome assessed in this data collection period.

^b^STI: sexually transmitted infection.

^c^STI tests include gonorrhea and chlamydia testing via throat and rectal swabs and urine and syphilis testing via a blood draw.

^d^PrEP: pre-exposure prophylaxis.

^e^Mitra blood microsampling will be performed only when participants report PrEP use before a study visit.

^f^—: outcome not assessed in this data collection period.

### RCT Randomization

Participants will be randomized 1:1:1 to the text intervention or WebApp intervention or control condition. Study staff will not be blinded to the condition that participants are randomized to. The randomization sequence will be stratified by city and use random permuted blocks of size 3.

### Follow-Up Evaluations

Follow-up evaluations will occur at 3, 6, and 9 months postenrollment. The data collection schedule is presented in Table 2. Participants in the text or the WebApp conditions who do not reduce sexual risk behaviors or who self-report a recent STI diagnosis and do not initiate PrEP or adhere to PrEP will be rerandomized 2:1 into either additional eCoaching with their originally assigned intervention (ie, text+eCoaching or WebApp+eCoaching) or to remain in their originally assigned intervention without the addition of eCoaching. Those that are rerandomized to step up to additional eCoaching will meet the eCoach at the 3-month visit, immediately following the assessment, for an introductory visit and to schedule their first eCoaching session. The active intervention period ends after 180 days. To measure the sustained effects of the intervention, the final data collection period comes during the 9-month visit, 3 months after the end of the intervention period.

### Measures

Study outcome measures and the timing of their administration are provided in Table 2. The outcomes are described below.

#### Outcomes of Interest

1. Change in condomless intercourse events: participants will be asked to report the frequency of condom use during sex in the last 3 months (*never* to *always*) as well as during the last 3 sexual encounters.
2. Change in condomless intercourse events when high on drugs or alcohol: participants will be asked to report whether they or their partners used substances during the last 3 sexual encounters.
3. Change in condomless intercourse events during sex work: participants will be asked to report condom use and partner type for sexual encounters over the previous 3 months as well as during the last 3 sexual encounters.
4. PrEP adherence: participants will self-report PrEP medication uptake and adherence on the ACASI. PrEP adherence is measured by blood sample levels of tenofovir diphosphate and emtricitabine triphosphate (TFV-DP/FTC-TP) with blood concentrations consistent with >4 doses per week.
5. HIV seroconversion: HIV tests will be administered at each study visit. Reactive results after baseline will be recorded as a seroconversion.
6. Incident STIs: participants will be asked to self-report new STI diagnoses from the past 3 months on the ACASI and they will be tested for gonorrhea and chlamydia via throat and rectal swabs and urine and syphilis via a blood draw.

#### Secondary Outcome

Transgender syndemic health index: participants will self-report education, employment status, housing stability, history in the criminal justice system, health care utilization, gender congruence, stress and resilience, substance use, and mental health.

#### Demographic Factors

Common demographic factors will be collected, including date of birth, race and ethnicity, gender identity, sex assigned at birth, sexual identity, education, employment status, health insurance, family income, housing stability, and history in the criminal justice system.

#### Health Care Use

Participants will be asked to rate their own health and medical services they may have used, including primary health care location, recent hospitalization, access to a primary care provider, comfort in discussing sexual relationships and gender identity, and HIV and STI testing. PrEP knowledge and use will be assessed using the PrEP Motivational Cascade [44]. Barriers to PrEP uptake or use are assessed using a measure developed and adapted by the ATN [45]. Gender confirmation surgery, medical procedures, hormone therapy, needle hygiene, and other gender presentation enhancements will be assessed using the Los Angeles Transgender Health Survey [46].

#### Gender Congruence, Stress, and Resilience

Gender congruence (ie, the alignment of gender expression and identity) is measured using items from the National Health Behavior Survey [47]. The adolescent version of the Gender Minority Stress and Resiliency Scale is used to assess gender-related discrimination and victimization, internalized transphobia, and pride [48]. The experiences of racism are captured using the Adolescent Discrimination Distress Index [49].

#### Substance Use

Substance use will be assessed via a urine screen to assess amphetamines, methamphetamines, cocaine, marijuana, and opiates using a generic 5-panel screening test (model HDOA-254; Confirm BioSciences) and an adapted version of the National Institute on Drug Abuse-modified alcohol, smoking, and substance involvement screening test [50].

#### Mental Health

Depression symptoms will be assessed using the 8-item Patient Health Questionnaire (PHQ-8) [51]. Participants will first be asked the first 2 items for the PHQ-8; those who report having some depressive symptoms (≥3 across the 2 PHQ items) will be asked to complete the remaining items of the scale.

#### Intimate Partner Violence

Experiences of relationship violence, including sexual assault, physical violence, isolation, privacy, and financial violations, are assessed [52,53]. Participants will be asked if they or their partners shared intimate photos of the other without permission [54].

#### Sexual Behavior

Sexual behavior will be assessed by asking whether they have engaged in vaginal, anal, or oral sex in the past 3 months. If they reported having sex in the past 3 months, participants will be asked how many main, casual, and exchange partners they had in the past 3 months, and how frequently (from none of the time to all of the time) they used a condom during insertive and receptive anal sex and vaginal sex. Participants are then asked about their 3 most recent sexual encounters and are asked to report on the number, genders, and types (ie, main, casual, exchange) of partners, sexual positioning, condom use, HIV status of partners, partner PrEP use, viral suppression of HIV-positive partners, and substance use by either the participant or their partners before or during sex [55].

#### Technology Adoption and Use

Technology use questions and items assessing participants’ attitudes toward technology were taken from items developed by the Pew Research Center’s Internet, Science, and Tech initiative. Participants are asked to report device ownership and operating system; how they access the internet; how they pay for service; how many hours a day they spend on the internet; how often they use mobile apps; frequency of internet use for social, sex-seeking, work, and health-seeking activities; and whether and how they may have faced discrimination when looking for partners on the web. In addition, the 8-item eHealth Literacy Scale will be used to assess participants’ perceptions of their skills for using the internet for health [56].

#### Ease of Use, Acceptability, and Satisfaction of the Intervention

Participants in the WebApp condition will be asked to rate the ease of use of their activities at the 3- and 6-month follow-up visits using the System Usability Scale (SUS) [57]. The SUS is a 10-item measure that asks participants to rate on a 1 (strongly disagree) to 5 (strongly agree) scale how much they agree with statements about the ease with which they were able to navigate the WebApp intervention. Participants in the text messaging condition will be asked at the 3- and 6-month follow-up visit the proportion of *TechStep* text messages they read. Participants in all conditions (including the information-only website condition) will be asked to answer questions on information quality, perceived usefulness of the information, and overall satisfaction with the intervention. We will also ask participants to rate their respective intervention on information quality and usefulness using items adapted from Horvath et al [58]. Finally, we will collect qualitative data on youth experiences by asking participants to state the 2 things they like most and least about *TechStep*.

#### User Engagement for the WebApp Intervention Arm

The WebApp intervention uses data collected during the active trial period to assess user engagement with the intervention. Standard use data include (1) log-in date and time, (2) type of device used, and (3) total duration of the session. Intervention use data for each participant will include the following variables reflecting peer-to-peer interaction: (1) date and content of original posts and (2) the number and content of replies to the original post. Additional user engagement variables collected are (1) frequency of posts; (2) number of comments; (3) number of tips viewed or favorited; (4) number of tracking behaviors established, frequency of tracking, and for each tracked behavior, the frequency of endorsing that behavior; (5) frequency of resource locator use and subtypes of resources sought; (6) total number of active intervention days; (7) number of times the participant updated their outward-facing profile features; and (8) total points earned.

### Data Analysis

Individuals’ baseline characteristics will be summarized by randomization arm using appropriate measures of central tendency and variability. We define intervention effects based on the difference in the average cumulative number (for count-valued outcomes such as instances of condomless anal intercourse) or proportion (for binary-valued outcomes such as PrEP uptake) of outcomes observed over 9 months of follow-up comparing a given active condition (eg, text messaging or WebApp) versus the info condition. We will estimate these effects using longitudinal targeted minimum loss-based estimation (LTMLE) [59-61]. LTMLE is a robust and efficient method for estimating treatment effects in the present context. First, it appropriately accounts for the study design and the fact that only some participants are eligible for rerandomization into the eCoaching intervention. Second, the method accounts for predictive and prognostic time-varying participant-level covariates, thereby increasing the power to detect intervention effects [62]. Finally, the method accounts for possibly informative participant dropout, which can reduce bias in effect estimates [63].

Implementation of LTMLE involves fitting several prespecified models that adjust for participant-level information. First, a sequence of outcome regressions is fit to model the average outcome (eg, average number of condomless anal intercourse events) at each time point, adjusting for baseline and time-varying covariates. Second, a regression is fit to model the cumulative probability of participant dropout, adjusting for baseline and time-varying covariates. These models will be fit using the super learner, a cross-validation–based technique for estimator selection [64]. This method aggregates results from a library of candidate regression estimators to build the most powerful predictor given the data at hand. In large samples, the method is guaranteed to perform as well as the unknown best-performing regression in the library. The method has also been validated in smaller samples via extensive simulation studies [65].

We will use level 0.05 Wald tests for each hypothesis using influence function-based standard error estimates. More information on this approach can be found in the paper by Benkeser et al [66].

### Power and Sample Size

The sample size was determined using Monte Carlo simulations. We designed a program to simulate trial data and calibrate the simulation to an existing data source to determine the appropriate distributions of risk behaviors in the study population. We evaluated the power to detect treatment effects across a range of sample sizes, intervention effect sizes, levels of missingness, strength of prognostic measurements in predicting outcomes, and proportion of participants who become eligible for rerandomization. Overall, we found that there would be >80% power to detect a difference of about 1.5 events of risky sexual behavior between the 2 intervention arms with 250 participants enrolled in the trial.

## Results

Funding for TechStep was awarded in June 2017, and phase 1 was approved by the IRB in April 2018. Phase 1 of the study was completed in December 2018. Initial phase 2 IRB approval came in June 2019. Data collection for phase 2 began in August 2019 and is expected to be completed in April 2021. As of March 2020, 54 participants have been enrolled in phase 2 of TechStep. Final results are anticipated in May 2021.

## Discussion

*TechStep* was designed to evaluate the efficacy of technology-based interventions for reducing HIV sexual risk behaviors and increasing PrEP initiation, adherence, and persistence among HIV-negative trans feminine, trans masculine, and gender nonconforming youth and young adults at risk of HIV and aims to improve their HIV and sexual health outcomes by providing culturally responsive, technology-based interventions.

There are a number of challenges to the *TechStep* clinical trial. First, it will require a multipronged effort to meet our recruitment goals as trans youth and young adults may not be accessible through traditional health care clinics. To address this, we will leverage social media advertising in both general (eg, Facebook) and trans-specific (eg, trans-specific subreddits) sites as well as recruitment at trans community events. Second, we do not provide smartphones or other web-enabled devices to study participants, but rather require that participants own or have access to a web-enabled device. This may restrict participation by lower socioeconomic status trans youth and young adults. However, if successful, it will increase the potential for scale-up. However, given that nearly 95% of teens have access to a smartphone [67], we believe that we will be able to capture the majority of the target population for this study. Third, given the high rates of mental health concerns among trans youth and young adults [68], medical and psychological services must be available during the study period. We will implement SRV-specific protocols to assess and provide referrals to medical and psychological services in the event that a participant should report a need for these services or experience any adverse reactions resulting from study procedures. In addition, the coaches for the eCoaching component have a protocol for managing crises that may arise during the eCoaching sessions and referrals to location-specific mental health resources.

The multilevel intersecting factors that impact the health and well-being of trans youth and young adults will require an equally complex response to reach these communities. The interventions designed and tested within TechStep provide highly scalable methods for reaching trans youth both in highly resourced urban areas and potentially also in geographically isolated rural areas. TechStep is designed so that comparisons may be made between those who receive the technology interventions in the control condition as well as to assess the additional benefit of receiving eCoaching compared with the text messaging or WebApp intervention alone. Thus, the lessons learned in *TechStep* will provide a strong foundation for subsequent technology-facilitated interventions with trans youth and young adults, and elucidate for whom more intensive interventions are required to reduce their risk for HIV. Thus, we believe that these lessons will move the field forward in important ways to address the complex needs of trans youth.

## Appendix

Multimedia Appendix 1 TechStep WebApp Homepage.

Multimedia Appendix 2 TechStep WebApp Resource Locator.

## Abbreviations

ACASI: audio computer-assisted self-interview
ATN: Adolescent Medicine Trials Network
CBT: Cognitive Behavioral Therapy
eCoaching: electronic coaching
IMB: Information, Motivation, Behavior
IRB: Institutional Review Board
LGBT: lesbian, gay, bisexual, transgender
LTMLE: longitudinal targeted minimum loss-based estimation
MI: Motivational Interviewing
PHQ: Patient Health Questionnaire
PrEP: pre-exposure prophylaxis
RCT: randomized controlled trial
SRV: subject recruitment venue
STI: sexually transmitted infection
SUS: System Usability Scale
YAB: youth advisory board
