# Supplementary figures and images for: Technology-Based Stepped Care to Stem Transgender Adolescent Risk Transmission: Protocol for a Randomized Controlled Trial (TechStep)
Source: JMIR Res Protoc. 2020 Aug 13;9(8):e18326. doi: 10.2196/18326 (PMC7458064; doi:10.2196/18326)

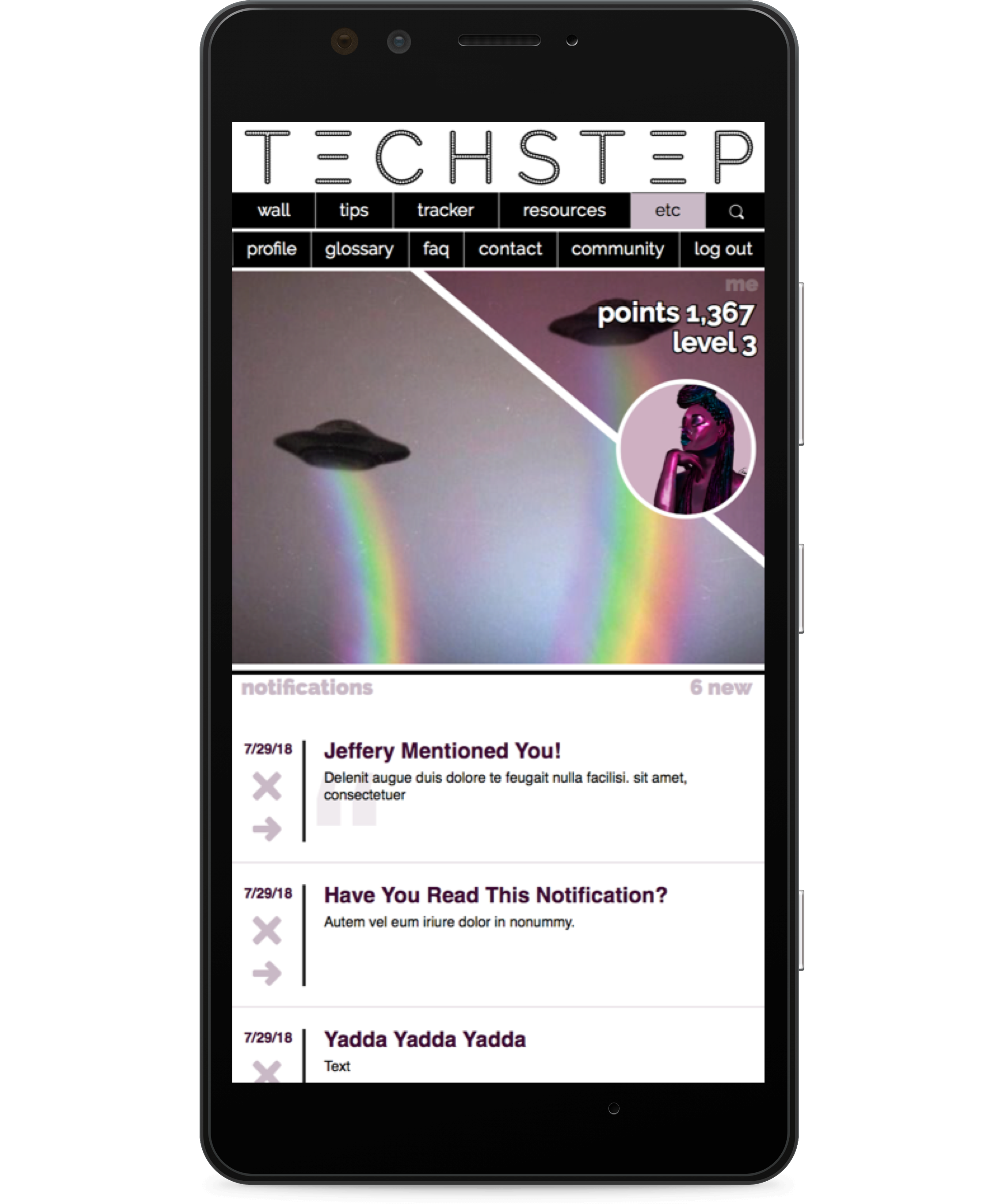

Supplement: Multimedia Appendix 1 [file resprot_v9i8e18326_app1.png]

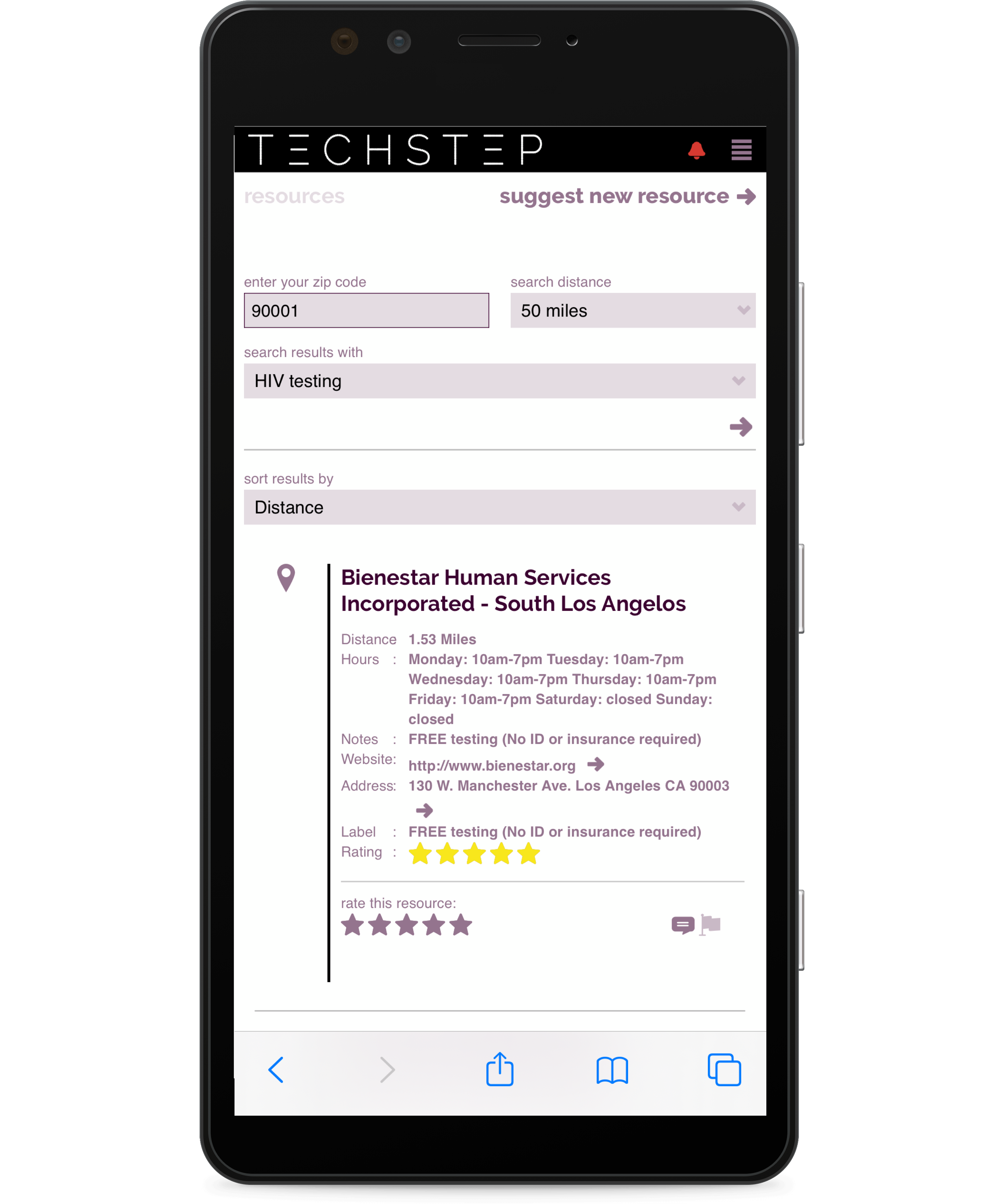

Supplement: Multimedia Appendix 2 [file resprot_v9i8e18326_app2.png]
